# Untargeted metabolomic profiling of fresh and dried leaf extracts of young and mature *Eucalyptus globulus* trees indicates differences in the presence of specialized metabolites

**DOI:** 10.3389/fpls.2022.986197

**Published:** 2022-11-14

**Authors:** Mafalda Pinto, Cristiano Soares, Ruth Pereira, José António Rodrigues, Fernanda Fidalgo, Inês Maria Valente

**Affiliations:** ^1^ GreenUPorto - Sustainable Agrifood Production Research Centre/INOV4AGRO, Departamento de Biologia, Faculdade de Ciências, Universidade do Porto, Porto, Portugal; ^2^ REQUIMTE, LAQV, Departamento de Química e Bioquímica, Faculdade de Ciências, Universidade do Porto, Porto, Portugal; ^3^ REQUIMTE, LAQV, ICBAS, Instituto de Ciências Biomédicas Abel Salazar, Universidade do Porto, Porto, Portugal

**Keywords:** allelopathy, GC-MS, LC-MS, Myrtaceae, phytochemicals, volatile compounds

## Abstract

Aqueous extracts from *Eucalyptus globulus* leaves contain a wide variety of specialized metabolites, mainly polyphenols and appreciable amounts of volatile compounds, which are responsible for their diverse biological activities, such as antioxidant, antimicrobial, and allelopathic features. For this reason, several studies have been conducted to explore the composition of *E. globulus* leaf extracts for multiple therapeutic and commercial applications. However, so far, the available bibliographic reports only refer to the chemical composition of extracts prepared with leaves from mature trees, leaving much to clarify about the composition of juvenile eucalyptus leaf extracts. Furthermore, there is no consensus regarding the type of leaves, fresh or dried ones, to be used in the extraction procedure, considering the highest recovery of biologically active compounds. In this sense, this study aimed to characterize the chemical composition of aqueous extracts prepared with fresh and dried leaves from young and mature *E. globulus* trees. For this, leaf biomass from young and mature *E. globulus* trees was collected in three distinct places from a forest area, and after oven-drying a portion of the leaves, an extraction in hot water was carried out, followed by GC-MS and HPLC-MS/MS analyses. The results revealed that the maturity of eucalyptus trees and biomass drying significantly influenced the volatile and non-volatile composition of the aqueous extracts. Accordingly, while fresh leaf extracts of young trees had great levels of hydrolysable tannins, extracts prepared with fresh leaves from mature trees presented a wide range of terpenes. When dried leaf material was used, extracts had notorious contents of amino acids derivatives, C_13_ norisoprenoids, fatty and other organic acids. Overall, this study showed, for the first time, that plant maturity (young *vs* mature) and pre-processing (fresh *vs* dried) of foliar biomass of *E. globulus* trees need to be considered in the preparation of leaf aqueous extracts depending on the desired purposes, since major changes in what regards biologically active compounds were found.

## 1 Introduction

*Eucalyptus globulus* Labill. subsp. *globulus*, also known as common eucalyptus or Tasmanian blue gum, is a perennial hardwood tree, belonging to the Myrtaceae family and native from southeast Australia (Catry et al., 2015). Due to its fast growth rate, wood characteristics, and high adaptation to different environmental conditions, such as water- and nutrient-deficient soils, this species was quickly widespread throughout the world, after its intentional introduction in some regions mainly for industrial pulpwood production (Catry et al., 2013; Catry et al., 2015).

The leaves of mature *E. globulus* trees are rich in essential oils with multiple beneficial properties for human health and have been widely used for many decades as air purifiers in the treatment of some respiratory tract diseases, such as asthma and bronchitis, sore throats, colds and coughs, and for muscle relaxation after intense physical activities (Hayat et al., 2015). Due to these numerous therapeutic properties, the composition of eucalyptus essential oils has been extensively studied by several authors (Silvestre et al., 1994; Song et al., 2009; Vilela et al., 2009; Daroui-Mokaddem et al., 2010; Mulyaningsih et al., 2010; Yong et al., 2019). In general, the essential oils of *E. globulus* are composed of a plethora of metabolites belonging to multiple chemical classes, like oxygenated monoterpenes as it is the case of 1,8-cineole (eucalyptol), listed as the most abundant compound in leaves, terpinen-4-ol, α-terpineol, and *ρ*-cymen-8-ol (Silvestre et al., 1994; Pombal et al., 2014; Morsi and Abdelmigid, 2016). Moreover, the volatile fraction of *E. globulus* leaves also comprise monoterpene hydrocarbons like α-pinene, camphene, α-terpinene, and limonene, and, in smaller amounts, some sesquiterpenes, such as aromadendrene and *β*-caryophyllene (Silvestre et al., 1994; Pombal et al., 2014; Morsi and Abdelmigid, 2016).

As specialized metabolites, these compounds may exert effects on other organisms, such as viruses, bacteria, fungi, and insects (Hayat et al., 2015; Horwath and Paul, 2015), thus detaining anti-viral, anti-bacterial, anti-fungal, and repellent activities, respectively (Choi et al., 2002; Sukontason et al., 2004; Rakotonirainy and Lavédrine, 2005; Nerio et al., 2010; Hayat et al., 2015; Roselló et al., 2015). Besides these widely recognized biological activities, volatile terpene compounds can be released into the environment, affecting the presence and development of surrounding plants, in a process known as allelopathy (May and Ash, 1990; Singh et al., 1991; Reigosa et al., 2000). This can be of particular interest when applied to weed control in a sustainable agriculture context (Pinto et al., 2021). Additionally, eucalyptus leaves detain several phenolic compounds such as ellagitannins like ellagic acid, hydroxycinnamic and hydroxybenzoic acids, flavonols like rutin, quercetin, catechin, and kaempferol 3-O-glucoside, as well as appreciable amounts of phenolic acids as it is the case of gallic and benzoic acids (Boulekbache-Makhlouf et al., 2013; Puig et al., 2018). These compounds have powerful reducing properties, mainly responsible for the antioxidant activity of *E. globulus* leaf extracts (Boulekbache-Makhlouf et al., 2013; Puig et al., 2018).

Although the chemical composition of the leaves from mature *E. globulus* trees is well studied, the phytochemical composition of juvenile leaves from young eucalyptus trees remain scarce and needs further research. In fact, leaves from young *E. globulus* trees are morphologically distinct from the mature ones: while leaves from young trees have a blue-grey color and thin and broad shape, mature trees’ leaves are characteristically green, thick, and narrow (James and Bell, 2001). These morphological differences suggest that there may be variations in their chemical composition. Actually, studies carried out with the aim of comparing the essential oil composition between juvenile and mature eucalyptus leaves revealed that the developmental stage influences the chemical composition of eucalyptus leaves (Silvestre et al., 1997; Russo et al., 2015). Essential oils from young *E. globulus* trees have higher monoterpenes’ contents, whereas mature tree leaves have higher oxygenated terpene levels (Silvestre et al., 1997). Also, Silvestre et al. (1997) and Russo et al. (2015) reported that juvenile eucalyptus leaves yield a greater amount of essential oils than mature *E. globulus* trees’ leaves; on the other hand, mature leaf essential oils have greater amounts of eucalyptol than the essential oils of juvenile eucalyptus leaves.

As an aromatic plant, *E. globulus* essential oils have received a lot of attention from researchers all over the world (reviewed in Hayat et al., 2015). However, hydrodistillation, the method by which essential oils are mainly obtained, can only extract volatile compounds (Dilworth et al., 2017). In contrast, the aqueous extraction usually obtained by decoction in hot water (Puig et al., 2018; Verdeguer Sancho et al., 2018), aside from extracting some volatile compounds, is also able to recover non-volatile compounds, such as phenolics, from eucalyptus leaves (Amakura et al., 2002). For this reason, the phytochemical composition of *E. globulus* leaf aqueous extracts has been receiving increasing attention in the last decade (Dudonne et al., 2009; Puig et al., 2018; Verdeguer Sancho et al., 2018). Furthermore, in a commercial context, aqueous extracts can be more attractive: while the production of essential oils has a very low yield and requires specialized equipment and professionals (Dilworth et al., 2017), aqueous extracts are easily produced with simple equipment and less technical expertise, and the final yield is much higher, as it can be seen by the simple procedures employed by other authors (Dudonne et al., 2009; Puig et al., 2018; Verdeguer Sancho et al., 2018). Thus, the discovery of natural sources of allelopathic compounds and their presence in formulations easy to obtain, with no dependence on industrial equipment, might represent the right step in future farming to reduce the negative footprint of synthetic herbicides.

Up to now, research has been mainly focused on mature leaves (Amakura et al., 2009; Boulekbache-Makhlouf et al., 2013; Puig et al., 2018). Due to their greater proximity to the ground, young eucalyptus trees tend to be more susceptible to herbivore attack than mature trees and, thus, young foliage is generally richer in defensive compounds than mature foliage (O’Reilly-Wapstra et al., 2007). In this way, the composition of the leaves from young plants and the proportion in which the compounds are found may be different from the leaves of mature trees. Moreover, the use of the leaves from young eucalyptus trees, instead of the old ones, can represent a sustainable strategy for controlling the dispersion of eucalyptus, outside managed plantations. Indeed, only at later developmental stages appears the first reproductive structures and then the production of the fruits, the capsules, that are responsible for seed dispersal (Calviño-Cancela and Rubido-Bará, 2013). Furthermore, due to their fire-adaptation characteristics, *E. globulus* trees rapidly regenerate after a wildfire, mainly by the resprouting from dormant buds, which lead to the fast spreading of this species even over areas previously inhabited by native species, hampering the post-fire management of areas recently affected by wildfires (Catry et al., 2013).

Thus, bearing in mind the greater recovery of compounds with potential allelopathic activities that can be further explored as biocides, this study aims to compare, for the first time, the chemical composition of aqueous extracts prepared from juvenile and mature *E. globulus* leaves, through an extensive characterization of their volatile and polyphenolic composition. Additionally, the effect of leaf processing (fresh vs dried leaves) on the phytochemical profile will also be assessed.

## 2 Materials and methods

### 2.1 Chemicals

Methanol (HPLC gradient grade, Panreac, Barcelona, Spain) and formic acid (99%, ChemLab, Zedelgem, Belgium) were used for chromatographic mobile phase preparation and extracts dilution. High purity water (resistivity higher than 18.2 MΩ cm) from a Direct-Q 3UV water purification system (Millipore Iberia, Madrid, Spain) was used for the preparation of solutions and chromatographic mobile phase. Dichloromethane (analytical grade) was purchased from Chem-Lab. Mix cannabis terpenes standards in methanol (products references CAN-TERP-MIX1 and CAN-TERP-MIX1; purities comprised in the range 75-99.5%) for identification of gas chromatography mass spectrometry (GC-MS) peaks were purchased from SpexCertiPrep, Inc. (Stanmore, UK).

### 2.2 Aqueous extracts – experimental design and preparation procedure

**Figure 1:**
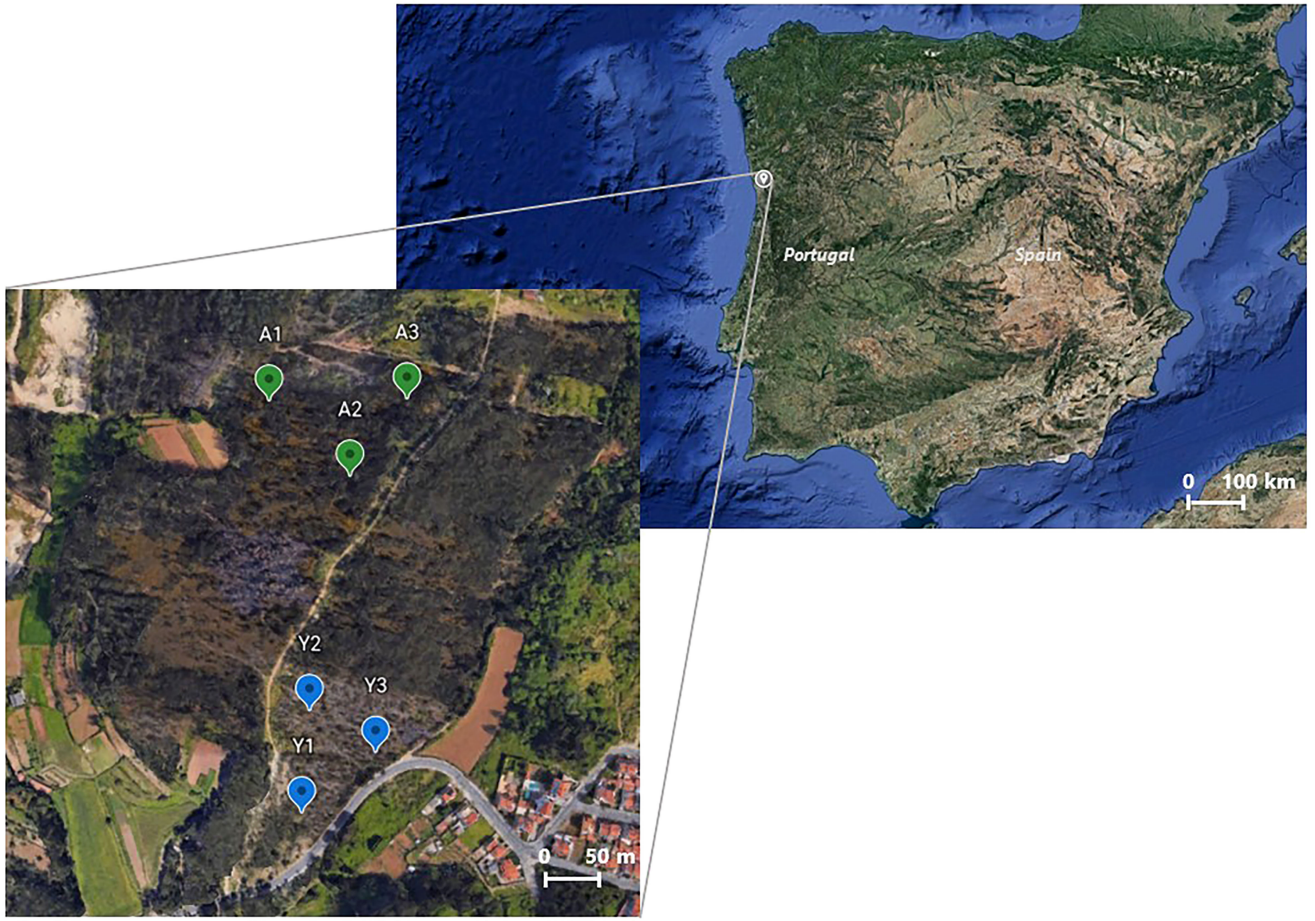
Sites of a burnt forest area where young (Y; Y1, Y2, and Y3) and mature (A; A1, A2, and A3) *E. globulus* leaves were harvested in February 2021. Retrieved from: Google Earth.

Branches of young and mature *E. globulus* Labill. trees were randomly collected in February 2021 (winter season) in three distinct places of a forest area burnt in July 2020 (Porto, Portugal: 41.193347, -8.529286), as illustrated in **Figure 1**. The criterion used to categorize the trees into young and mature was based not only on height but mainly on the color and morphology of the eucalyptus leaves. Thus, the young trees considered in this study comprised exclusively branches with blue-grey leaves, morphologically wide and thin; while the adult trees were composed of branches with green leaves with the characteristic morphology: narrow, pointed, and thick. Trees comprising branches with transitional leaves were not considered in this work. Upon collection, the fresh eucalyptus leaves were manually detached and randomly assigned into two different portions. One of these portions was immediately oven-dried at 60°C until reaching a constant weight. Then, fragments of fresh and dried leaves from young and mature trees collected in the three different sites (i.e., Y1, Y2, Y3, A1, A2, and A3 from **Figure 1**) suffered an extraction procedure in deionized water at 70°C, for 30 min, according to the methodology of Pinto et al. (2021). Afterwards, the twelve obtained extracts underwent two successive centrifugations (-4°C, 15 000 g) for 25 and 15 min, respectively. Given that leaves’ moisture content affects the final concentration of the extracts, the water content of the eucalyptus leaves was calculated (young trees: 62 ± 1.7% m/m; mature trees: 57 ± 1.1% m/m) and used to adjust the concentration of the extracts prepared from fresh leaves. Thus, the dried leaf extracts of young and mature eucalyptus trees were prepared for a final concentration of 250 g_dry weight_ L^-1^, whereas the fresh leaves’ extracts of young trees were prepared at 617 g_fresh weight_ L^-1^ and the aqueous extract prepared with fresh leaves of mature trees had a final concentration of 556 g_fresh weight_ L^-1^. Finally, the twelve solutions were filtered through 1.2 μm nitrocellulose filters and stored at -80 °C until use.

### 2.3 GC-MS analysis

Sample preparation of the extracts was performed using liquid-liquid extraction for the extraction of the compounds to dichloromethane, based on the protocol of Cabredo-Pinillos et al. (2006). In centrifuge glass tubes, 5 mL of the aqueous leaf extracts, 0.8 g of NaCl and 1 mL of dichloromethane (containing 20 mg L^-1^ of 3-octanol as internal standard) were added. The mixture was vortexed and extracted in an ultrasonic bath at room temperature (~20 °C) for 15 min. The tubes were centrifuged (Z 300K, Hermle, Germany) at 2500 rpm for 5 min. The aqueous phase was discarded, and the organic extract was carefully collected with a Pasteur pipette to a microcentrifuge tube for drying with anhydrous sodium sulphate. The dried extract was collected to a glass vial for GC-MS analysis. Each sample was extracted in duplicate.

The GC-MS analysis was performed in a Thermo Scientific Trace 1300, ISQ Single Quadrupole MS system using a TraceGOLD™ TG-5MS (30 m 0.25 mm; 0.25 μm). The oven temperature was maintained at 40 °C for 2 min, raised to 250°C at a rate of 3°C min^-1^, and kept steady at 250°C for additional 5 min. Helium was the carrier gas at a flow rate of 1 mL min^-1^. A sample of 1 μL of the dichloromethane extract was injected in split mode (1:20) in the injector kept at 250 °C. The electron-impact ionisation (EI) MS detector was operated at 250 °C and 70 eV in the range m/z 40 to 350. The data acquisition was carried out using XCalibur software version 2.2 (Thermo Electron Corporation).

### 2.4 High performance liquid chromatography and tandem mass spectrometry (HPLC-MS/MS) analysis

Samples of all aqueous extracts were filtered through 0.45 µm regenerated cellulose syringe filters (Sartorius, Germany) and diluted (1:2) in a mixture (1:1, v/v) composed of methanol and 0.1% (v/v) aqueous formic acid for further analysis by HPLC-MS/MS, in duplicate.

The characterization of the extracts was performed in a high-resolution mass spectrometer attached to a liquid chromatography system (Vanquish Core, Thermo Scientific, Waltham, MA) with HESI source (Orbitrap Exploris 120 Mass Spectrometer, Thermo Scientific). A Gemini C_18_ column (150 mm × 4.6 mm; 3 μm particle size) and a guard column (4 mm × 3.0 mm) from Phenomenex (Torrance, CA) were used at 29°C. The chromatographic conditions for the separation of the compounds and the injection volume were those described by Valente et al. (2018). The MS analysis was performed in the negative ionization mode. The operating conditions used in the MS detector as well as the chromatographic separation conditions are described in the Supplementary information (**Table S1**). Data acquisition was done using Thermo Scientific Chromeleon Chromatography Data System (Thermo Electron Corporation, Waltham, MA).

### 2.5 Processing of raw HPLC-MS and GC-MS data using MZmine

The RAW files obtained HPLC-MS/MS analyses were extracted and converted to mzML files using the ProteoWizard MSconvert tool (Kessner et al., 2008) and subsequently processed using MZmine 2.53 (Pluskal et al., 2010).

For GC-MS data, the noise level for mass detection was set to 1x10^3^. The ADAP Chromatogram Builder Module (Myers et al., 2017) was used for the chromatogram builder (5 scans as minimum group size; group intensity threshold 1000; minimum highest intensity 1000; m/z tolerance 0.75 m/z). The ADAP Chromatogram Deconvolution Module (Myers et al., 2017) was used with the following settings: S/N threshold 7; S/N estimator: Wavelets coefficient SN; minimum feature height 100; coefficient/area threshold: 30; peak duration range: 0.01 – 1; RT wavelet range: 0.01 – 0.1; m/z centre calculation: median. The multivariate curve resolution was used for spectral deconvolution, considering 0.2 min as deconvolution window width, 0.1 min as retention time tolerance, and a minimum number of peaks of 2. The chromatograms were aligned using the ADAP aligner with the following parameters: 0.17 as minimum confidence tolerance; 0.2 min as retention time tolerance; 0.1 m/z as m/z tolerance; score threshold of 0.75; score weight of 0.1; retention time similarity given by difference. The peak list was gap-filled with the peak finder (multithreaded) module (intensity tolerance at 0.05%, m/z tolerance at 5 ppm, and absolute RT tolerance of 0.05 min). Finally, the peak areas were normalized using the peak area of the internal standard (3-octanol). The identification of the compounds was performed by comparing the obtained mass spectra with the National Institute of Standards and Technology (NIST) mass spectra database (version NIST 17) and by comparison with mass spectra of terpenes’ standards (section 2.1).

For HPLC-MS data, mass detection was performed both in MS1 level (noise level set at 5x10^6^) and MS2 level (noise level set at 5x10^4^). The ADAP Chromatogram Builder Module (Myers et al., 2017) was used for the chromatogram builder using MS1 (5 scans as minimum group size; group intensity threshold 1.5x10^7^; minimum highest intensity 1.5x10^7^; m/z tolerance 5 ppm). The ADAP Chromatogram Deconvolution Module (Myers et al., 2017) was used with the following settings: S/N threshold 7; S/N estimator: Intensity window SN; minimum feature height 1x10^6^; coefficient/area threshold: 50; peak duration range: 0.01 – 2; RT wavelet range: 0.01 – 0.5; m/z centre calculation: auto; m/z range for MS2 scan pairing (Da): 0.025; RT range for MS2 scan pairing (min): 0.15. The chromatograms were deisotoped using the isotopic peaks grouper algorithm with a m/z tolerance of 0.01 and a RT (retention time) tolerance of 0.1 min. Peak alignment was achieved by join the aligner using m/z tolerance of 5 ppm (weight for m/z: 75) and retention time tolerance of 0.5 min (weight for retention time: 25). The peak list was gap-filled with the peak finder (multithreaded) module (intensity tolerance at 0.05%, m/z tolerance at 5 ppm, and absolute RT tolerance of 0.1 min). The final peaks list was filtered considering a minimum of 2 peaks per row and keeping only the features with MS2 scan. Adducts, complexes, and fragment ions were also removed from the peaks list. The compounds identification was performed by comparison of MS spectra with pure standards and literature information.

The resulting peak lists for both GC-MS and HPLC-MS analyses were exported as.csv files for statistical analysis.

### 2.6 Multivariate statistical analysis

Each aqueous extract comprised three independent replicates (n = 3), and each sample was analyzed in duplicate (technical replicates). The peak intensity table generated by MZmine was uploaded to MetaboAnalyst 5.0 (Pang et al., 2021) for statistical analysis. Missing values were replaced with the detected minimum value for statistical analysis, assuming that they were below the limits of instrument detection sensitivity during the data integrity check. Data were filtered considering the interquartile range (IQR) to eliminate variables that are near-constant values throughout the experiment and were log-transformed. Principal component analysis (PCA) was performed, and the groups were displayed in the plots considering 95% confidence regions. A two-way analysis of variance (ANOVA) was applied to the results; the significance threshold is defined as the corrected *p*-value < 0.05 and the False Discovery Rate is chosen for multiple testing correction.

The metabolic alterations caused by the leaf pre-processing and the maturity of the plant were evaluated sing MetaboAnalyst 5.0. Univariate analysis (Volcano plots) was performed considering unequal variance between groups, fold change (FC) threshold of 2 and *p* < 0.05. The heatmap plot showing the metabolites affected by both leaves’ processing and maturity was obtained using normalized data and autoscale features, Euclidean distance measure, and Ward clustering method; only group averages were displayed.

## 3 Results

### 3.1 Metabolomic patterns in fresh and dried leaf extracts of young and mature *E. globulus*

**Figure 2:**
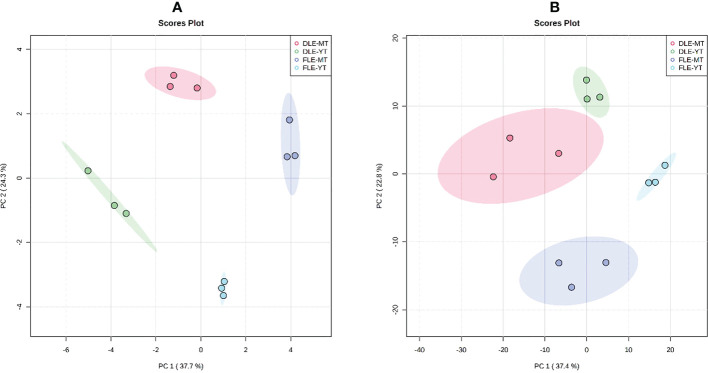
Principal component analysis (PCA) plots based on the results of **(A)** GC-MS and **(B)** HPLC-MS analysis of the 4 groups of samples: the aqueous extracts prepared with dried leaves of mature *E globulus* trees (DLE-MT; red); the aqueous extracts prepared with fresh leaves of mature *E globulus* trees (FLE-MT; purple); the aqueous extracts prepared with dried leaves of young *E globulus* trees (DLE-YT; green); and the aqueous extracts prepared with fresh leaves of young *E globulus* trees (FLE-YT; blue). Scree plots are shown in **Figure S1**.

To evaluate the effect of plant maturity and leaf pre-processing method (fresh or dried leaves) on the chemical composition of *E. globulus* leaf extracts, samples were subjected to metabolic profiling by GC-MS and HPLC-MS. The metabolome of the aqueous extracts showed a total of 112 and 871 features in GC-MS and LC-MS analysis, respectively, considering all the samples together. The PCA analysis, carried out to assess the chemometric separation among groups of samples, revealed a complete separation of the 4 groups (**Figure 2**): aqueous extracts prepared with fresh leaves of young *E. globulus* trees; aqueous extracts prepared with dried leaves of young *E. globulus* trees; aqueous extracts prepared with fresh leaves of mature *E. globulus* trees; aqueous extracts prepared with dried leaves of mature *E. globulus* trees.

**Figure 3:**
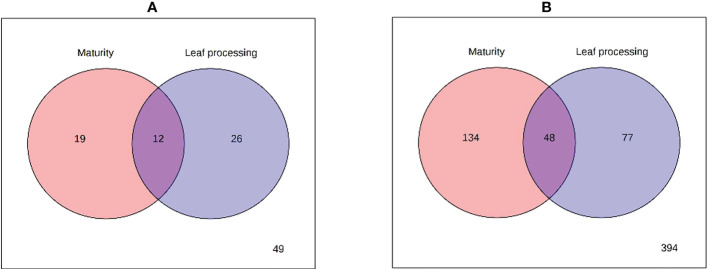
Venn diagrams of the results from two-way ANOVA of **(A)** GC-MS and **(B)** LC-MS analyses.

Since the PCA analysis cannot discriminate which factor (plant maturity or leaf pre-processing) was responsible for the observed group separation (**Figure 2**), a two-way ANOVA was performed comparing the levels of each metabolite in each group, and its outcomes are presented in the Venn diagrams of **Figure 3**. The results of the GC-MS characterization revealed that the levels of 19, 26, and 12 volatile metabolites were significantly affected by the plants’ maturity, the leaf pre-processing, and both factors, respectively (**Figure 3A**). A more complex profile was obtained by HPLC-MS analysis that resulted in 134, 77, and 48 metabolic features considerably altered by the plants’ maturity, the leaf pre-processing, and by both factors, respectively (**Figure 3B**).

From the compounds with statistically significant differences for the factors plant’s maturity and leaf pre-processing between the groups of samples, a total of 153 metabolites were tentatively identified (**Table S2**).

### 3.2 Metabolic alterations caused by the leaf pre-processing

**Figure 4:**
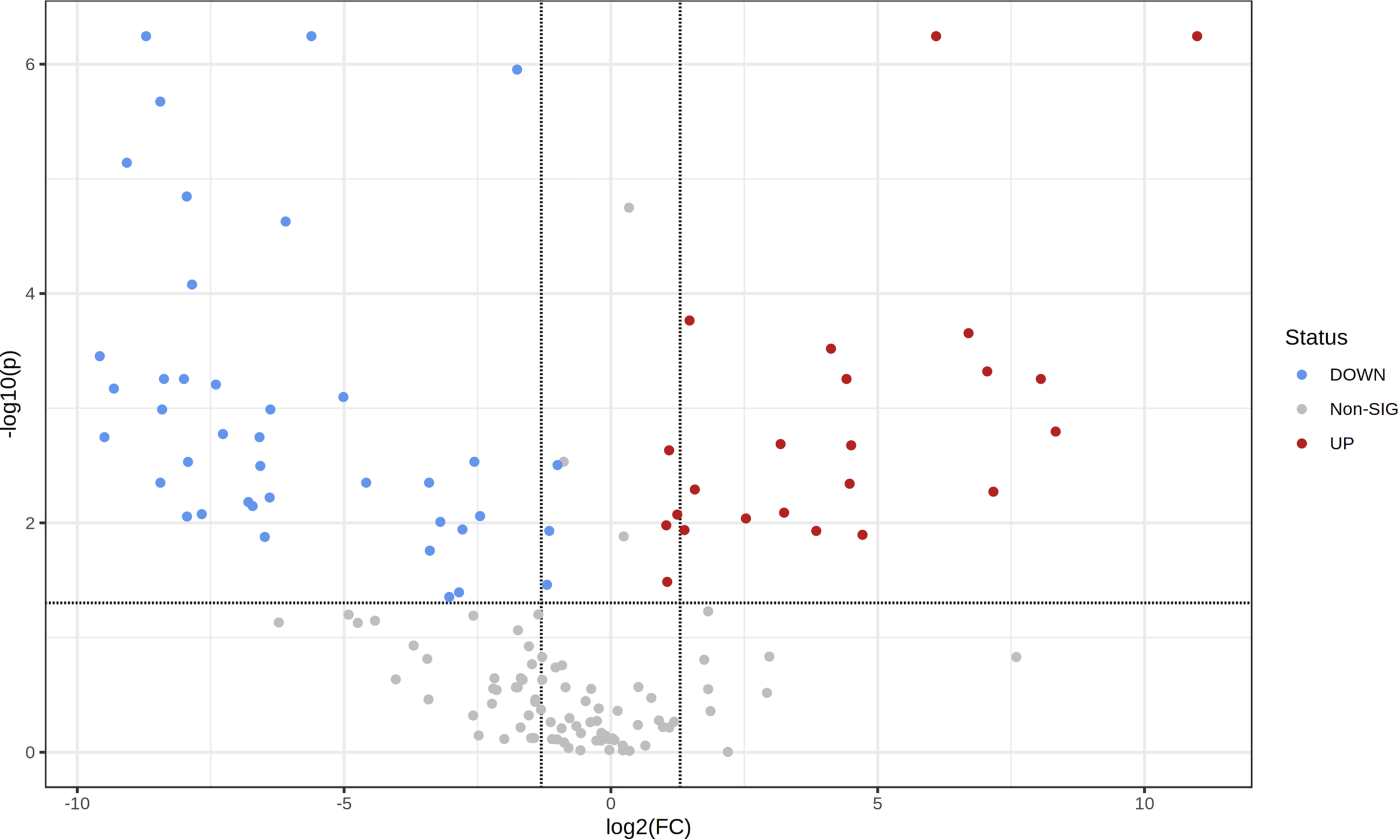
Volcano plot showing the metabolic features’ relative content in fresh leaf extracts (FLE) and dried leaf extracts (DLE). Higher content in FLE is given by FC < 2 and *p* < 0.05; higher content in DLE is given by FC > 2 and *p* < 0.05.

To assess the metabolic differences between leaf aqueous extracts resulting from the drying process, the levels of the 153 identified metabolites were compared between fresh and dried leaf extracts (**Table 1**). The volcano plot (**Figure 4**), resulting from the fold change analysis and the t-test, allowed the evaluation of the metabolites’ statistical significance between both groups of samples. The results revealed that the levels of 63 metabolites were significantly altered by the leaf pre-processing. Accordingly, it was also verified that regardless of the donor tree maturity, the pre-processing of the eucalyptus leaves was responsible for reducing the levels of 40 metabolites and forming or increasing the content of 23 compounds (**Table 1**, **Figure 4**).

**Table 1:** Results of the ANOVA test for the evaluation of the effect of the leaf pre-processing in extracts prepared with leaves from young and mature trees [*p* < 0.05; FC < -2: higher content in the fresh leaf extract (FLE); FC > 2: higher content in the dried leaf extract (DLE)].

Compound Name	Class	FC	*p* value
Glutamine	Amino acid or derivative	0.145	0.01
Methylserine hexoside	Amino acid or derivative	2027.700	<0.01
N-1-Deoxy-1-fructosyl)alanine	Amino acid or derivative	68.308	<0.01
Serine glucoside	Amino acid or derivative	104.150	<0.01
Dihydroxybenzoic acid pentoside derivative	Benzoic acid	0.004	<0.01
Protocatechuic acid	Benzoic acid	132.740	<0.01
3-Hydroxy-5, 6-epoxy-beta-ionone	C_13_ norisoprenoids	2.974	0.01
4-Hydroxy-4-(3-oxo-1-butenyl)-3,5,5-trimethylcyclohex-2-en-1-one	C_13_ norisoprenoids	5.777	0.01
Hexose polymer	Carbohydrate or derivative	0.020	<0.01
Melezitose or raffinose or maltotriose	Carbohydrate or derivative	0.170	<0.01
Monosaccharide IV	Carbohydrate or derivative	0.139	0.04
Oligosaccharide	Carbohydrate or derivative	0.015	<0.01
Turanose or palatinose or maltose or lactose or sucrose or trehalose	Carbohydrate or derivative	0.296	<0.01
(+)-Epigallocatechin	Condensed tannin	0.002	<0.01
(epi)catechin-(epi)catechingallate I	Condensed tannin	0.012	<0.01
(epi)catechin-(epi)gallocatechin	Condensed tannin	0.002	<0.01
Procyanidin B-type I	Condensed tannin	0.001	<0.01
Procyanidin B-type II	Condensed tannin	0.003	<0.01
Procyanidin B-type IV	Condensed tannin	0.002	<0.01
Procyanidin C-type	Condensed tannin	0.003	<0.01
2’,3’-Bis-O-degalloyl rugosin F isomer II	Ellagic acid or derivative	0.109	0.01
Digalloyl-HHDP-gluconic acid I	Ellagic acid or derivative	0.010	0.01
Digalloyl-HHDP-gluconic acid II	Ellagic acid or derivative	0.012	0.01
Digalloyl-HHDP-gluconic acid III	Ellagic acid or derivative	0.009	0.01
Digalloyl-HHDP-gluconic acid IV	Ellagic acid or derivative	0.011	<0.01
Digalloyl-HHDP-gluconic acid V	Ellagic acid or derivative	0.004	<0.01
Digalloyl-HHDP-gluconic acid VII	Ellagic acid or derivative	0.010	<0.01
Ellagic acid derivative I	Ellagic acid or derivative	0.001	<0.01
Ellagic acid derivative II	Ellagic acid or derivative	0.006	<0.01
Ellagic acid derivative III	Ellagic acid or derivative	0.003	<0.01
Eucalbanin C I	Ellagic acid or derivative	0.004	0.01
Glansrin D or degalloyl rugosin F isomer II	Ellagic acid or derivative	0.005	0.01
Hydrolysable tannin I	Ellagic acid or derivative	0.011	0.01
6,8-dihydroxy-octanoic acid	Fatty acid or derivative	9.061	<0.01
FA hydroxy (11:2/11:2) I	Fatty acid or derivative	2.078	0.03
FA hydroxy (11:2/11:2) III	Fatty acid or derivative	0.004	<0.01
Galactosylglycerol or Glucosylglycerol	Fatty acid or derivative	17.444	<0.01
Phloionolic acid	Fatty acid or derivative	14.403	0.01
Tetrahydroxyoctadecenoic acid	Fatty acid or derivative	22.227	<0.01
Catechin	Flavonoid	0.031	<0.01
Digalloylglucose I	Gallic acid or derivative	2.053	0.01
Digalloylglucose IV	Gallic acid or derivative	2.369	0.01
Digalloylglucose V	Gallic acid or derivative	22.665	<0.01
Gallic acid + CO2	Gallic acid or derivative	143.830	0.01
Gallic acid pentoside	Gallic acid or derivative	0.004	<0.01
Galloyl glucose derivative X	Gallic acid or derivative	0.094	<0.01
Galloylglucose I	Gallic acid or derivative	9.482	0.01
Galloylglucose II	Gallic acid or derivative	21.331	<0.01
cis-5-caffeoylquinic acid	Hydroxycinnamic acid	0.095	0.02
Coumaroyl hexose	Hydroxycinnamic acid	2.601	0.01
Sinapoyl glucoside I	Hydroxycinnamic acid	0.006	<0.01
trans-5-caffeoylquinic acid	Hydroxycinnamic acid	0.500	<0.01
eucalyptol	Monoterpenoid	0.436	0.03
2-hydroxypropanedioic acid	Organic acid	26.265	0.01
2-methylcitric acid or homocitric acid I	Organic acid	2.130	<0.01
Arabinaric acid	Organic acid	322.890	<0.01
Erythronic acid or Threonic acid I	Organic acid	2.778	<0.01
Erythronic acid or Threonic acid II	Organic acid	0.003	<0.01
Galactaric acid or Glucaric acid II	Organic acid	0.122	0.04
Tartaric acid or meso-tartaric acid II	Organic acid	266.390	<0.01
α-Limonene diepoxide	Terpenoid derivative	0.183	0.01
Cypellocarpin B	Terpenoid derivative	0.042	<0.01
exo-2-Hydroxycineole acetate	Terpenoid derivative	0.449	0.01

Regarding the chemical classes of the identified metabolites, fresh leaf extracts were richer in carbohydrates, condensed tannins, ellagitannins (ellagic acid derivatives), hydroxycinnamic acids, and terpenes than dried leaf extracts (FC < 2 and *p* < 0.05; **Table 1**). On the other hand, aqueous extracts prepared with dried leaves presented a higher content of amino acid derivatives, C_13_ norisoprenoids, fatty acid derivatives, and organic acids than fresh leaf extracts (FC > 2 and *p* < 0.05; **Table 1**). Benzoic acids have also showed to be affected by the pre-processing of *E. globulus* leaves; however, the metabolite present at higher levels in fresh leaf extracts are different from the one in aqueous extracts prepared with dried leaves. While fresh leaf extracts had great contents of dihydroxybenzoic acid pentoside derivative, extracts prepared with dried leaves detained considerable levels of protocatechuic acid (**Table 1**).

### 3.3 Metabolic alterations induced by the maturity of the tree

**Figure 5:**
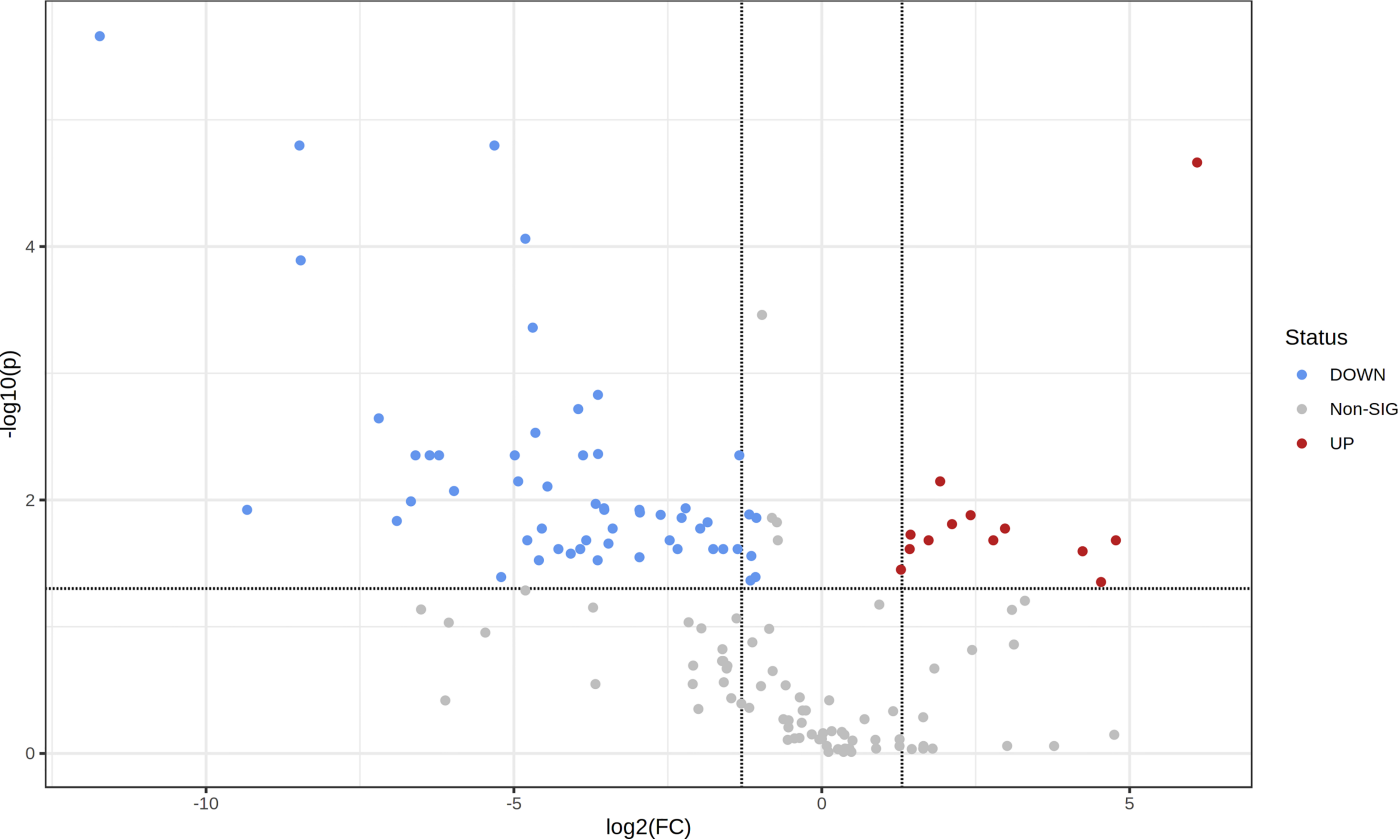
Volcano plot showing the metabolic features relative content in fresh leaf extracts (FLE) and dried leaf extracts (DLE). Higher content in young trees (YT) is given by FC < 2 and *p* < 0.05; higher content in mature trees (MT) is given by FC > 2 and *p* < 0.05.

A comparison between the metabolite concentration of aqueous extracts prepared with leaves from young and mature trees was carried out to understand the effects of *E. globulus* tree maturity on the chemical composition of the leaf extracts (**Table 2** and **Figure 5**). In this case, FC < 2 denotes greater contents of the phytochemical in juvenile leaf extracts and FC > 2 means that the metabolite was present in higher levels in mature leaf extracts. Based on **Table 2**, 68 metabolites were significantly altered by the factor “maturity of the tree”, with 55 compounds being more abundant in juvenile leaf extracts than in those from mature trees. In fact, the statistical analysis showed that this factor influenced more strongly the metabolite diversity of fresh leaf extracts than that of dried leaf extracts (**Figure 5**).

**Table 2:** Results of the ANOVA test for the evaluation of the effect of the trees’ maturity in fresh and dried *E. globulus* leaf extracts [*p* < 0.05; Fold change FC < -2: higher content in extracts prepared with leaves from young trees (YT); FC > 2: higher content in extracts prepared with leaves from mature trees (MT)].

Compound Name	Class	FC	*p* value
Methyl 3-(2,3-dihydroxy-3-methylbutyl)-4-hydroxybenzoate or isopropyl 2-hydroxy-3-(3-hydroxy-4-methoxyphenyl)propanoate or isopropyl 2-hydroxy-3-(4-hydroxy-3-methoxyphenyl)propanoate I	Benzoic acid	0.080	0.03
Methyl 3-(2,3-dihydroxy-3-methylbutyl)-4-hydroxybenzoate or isopropyl 2-hydroxy-3-(3-hydroxy-4-methoxyphenyl)propanoate or isopropyl 2-hydroxy-3-(4-hydroxy-3-methoxyphenyl)propanoate II	Benzoic acid	0.086	0.01
Methyl 3-(2,3-dihydroxy-3-methylbutyl)-4-hydroxybenzoate or isopropyl 2-hydroxy-3-(3-hydroxy-4-methoxyphenyl)propanoate or isopropyl 2-hydroxy-3-(4-hydroxy-3-methoxyphenyl)propanoate III	Benzoic acid	0.095	0.02
3-(2,3-Dihydroxypropoxy)-2-hydroxypropyl galactopyranoside	Carbohydrate or derivative	3.329	0.02
3-Deoxy-D-glycero-D-galacto-2-nonulosonic acid	Carbohydrate or derivative	0.395	<0.01
Sulphated carbohydrate	Carbohydrate or derivative	0.078	0.01
2’,3’-Bis-O-degalloyl rugosin F isomer I	Ellagic acid or derivative	0.052	0.02
Dehydro-galloyl-HHDP-hexoside	Ellagic acid or derivative	0.013	<0.01
di-HHDP-glucose IV	Ellagic acid or derivative	0.071	0.02
di-HHDP-glucose IX	Ellagic acid or derivative	0.064	<0.01
di-HHDP-glucose V	Ellagic acid or derivative	0.453	0.03
Ellagic acid I	Ellagic acid or derivative	0.448	0.04
Glansrin D or degalloyl rugosin F isomer I	Ellagic acid or derivative	0.008	0.01
HHDP digalloyl glucose II	Ellagic acid or derivative	0.027	0.04
HHDP galloyl glucose I	Ellagic acid or derivative	0.479	0.01
HHDP galloyl glucose III	Ellagic acid or derivative	0.442	0.01
HHDP galloyl glucose IV	Ellagic acid or derivative	0.295	0.02
HHDP galloyl glucose VII	Ellagic acid or derivative	0.041	0.03
Hydrolysable tannin II	Ellagic acid or derivative	18.837	0.03
tri-O-methylellagic acid	Ellagic acid or derivative	0.040	<0.01
(3S,4R)-3,4,5-trihydroxy-2-oxo-pentanoic acid	Fatty acid or derivative	0.216	0.01
9,12,13-trihydroxy-10,15-octadecadienoic acid	Fatty acid or derivative	0.128	0.03
DGGA(28:4;O)	Fatty acid or derivative	0.025	<0.01
Avicularin or guajavarin I	Flavonoid	0.043	0.02
Avicularin or guajavarin II	Flavonoid	0.003	<0.01
Kaempferol 3-glucuronide	Flavonoid	0.163	0.01
Kaempferol-3-O-rutinoside	Flavonoid	0.080	<0.01
Luteolin 7-O-glucuronide	Flavonoid	0.000	<0.01
Quercetin	Flavonoid	0.002	0.01
Quercetin-4’-O-glucoside or quercetin-3-glucoside (isoquercitrin)	Flavonoid	0.003	<0.01
Digalloylglucose II	Gallic acid or derivative	0.091	0.02
Digalloylglucose III	Gallic acid or derivative	0.086	0.01
Digalloylglucose VI	Gallic acid or derivative	0.081	<0.01
Gallic acid derivative	Gallic acid or derivative	0.180	0.02
Gallotannin	Gallic acid or derivative	27.404	0.02
Galloyl glucose derivative I	Gallic acid or derivative	2.438	0.04
Galloyl glucose derivative III	Gallic acid or derivative	6.896	0.02
Galloylshikimic acid I	Gallic acid or derivative	0.012	<0.01
Galloylshikimic acid II	Gallic acid or derivative	0.010	<0.01
Galloylshikimic acid III	Gallic acid or derivative	0.007	<0.01
Hydroxybenzoyl galloyl glucoside	Gallic acid or derivative	23.193	0.04
Tetragalloylglucose II	Gallic acid or derivative	0.059	0.03
Tetragalloylglucose III	Gallic acid or derivative	0.036	0.02
Tetragalloylglucose IV	Gallic acid or derivative	0.010	0.01
Trigalloylglucose I	Gallic acid or derivative	0.046	0.01
Trigalloylglucose II	Gallic acid or derivative	0.128	0.01
Trigalloylglucose III	Gallic acid or derivative	0.129	0.01
Trigalloylglucose V	Gallic acid or derivative	0.068	<0.01
Trigalloylglucose VI	Gallic acid or derivative	0.254	0.02
Trigalloylglucose VII	Gallic acid or derivative	0.276	0.01
cis-5-O-p-coumaroylquinic acid	Hydroxycinnamic acid	0.388	0.02
Mallophenol B or Macarangioside B III	Megastigmane derivative	2.716	0.02
α-terpineol	Monoterpenoid	0.474	0.04
Limonene-1,2-diol	Monoterpenoid	3.791	0.01
pinocarvone	Monoterpenoid	7.865	0.02
terpinen-4-ol	Monoterpenoid	0.330	0.02
trans-pinocarveol	Monoterpenoid	4.332	0.02
3-dehydroshikimic acid	Organic acid	0.032	<0.01
Aconitic acid I	Organic acid	0.016	0.01
Aconitic acid II	Organic acid	0.033	0.01
Azelaic acid or 3-methylsuberic acid	Organic acid	68.393	<0.01
Shikimic acid	Organic acid	0.039	<0.01
Tartaric acid or meso-tartaric acid I	Organic acid	2.691	0.02
Citrinin	Other compounds	0.066	0.02
α-terpinyl acetate	Terpenoid derivative	0.036	<0.01
Cypellocarpin C or Eucalmalduside A I	Terpenoid derivative	5.341	0.01
Resinoside A or Resinoside B	Terpenoid derivative	0.197	0.02
Triterpene acid-O-hexoside II	Terpenoid derivative	0.206	0.01

Although significant differences in the individual metabolite composition in young and mature trees were observed, in general extracts prepared with leaves from young trees were richer in benzoic acids, carbohydrates, ellagic acid or derivatives, fatty acid derivatives, flavonoids, gallic acid derivatives, hydroxycinnamic acids, and terpenoid derivatives (**Table 2**). Instead, extracts prepared with mature leaves presented higher levels of megastigmane derivative, monoterpenoids, and organic acids (**Table 2**).

### 3.4 Metabolomic alterations caused by the interaction between the leaf pre-processing and the maturity of the tree

**Figure 6:**
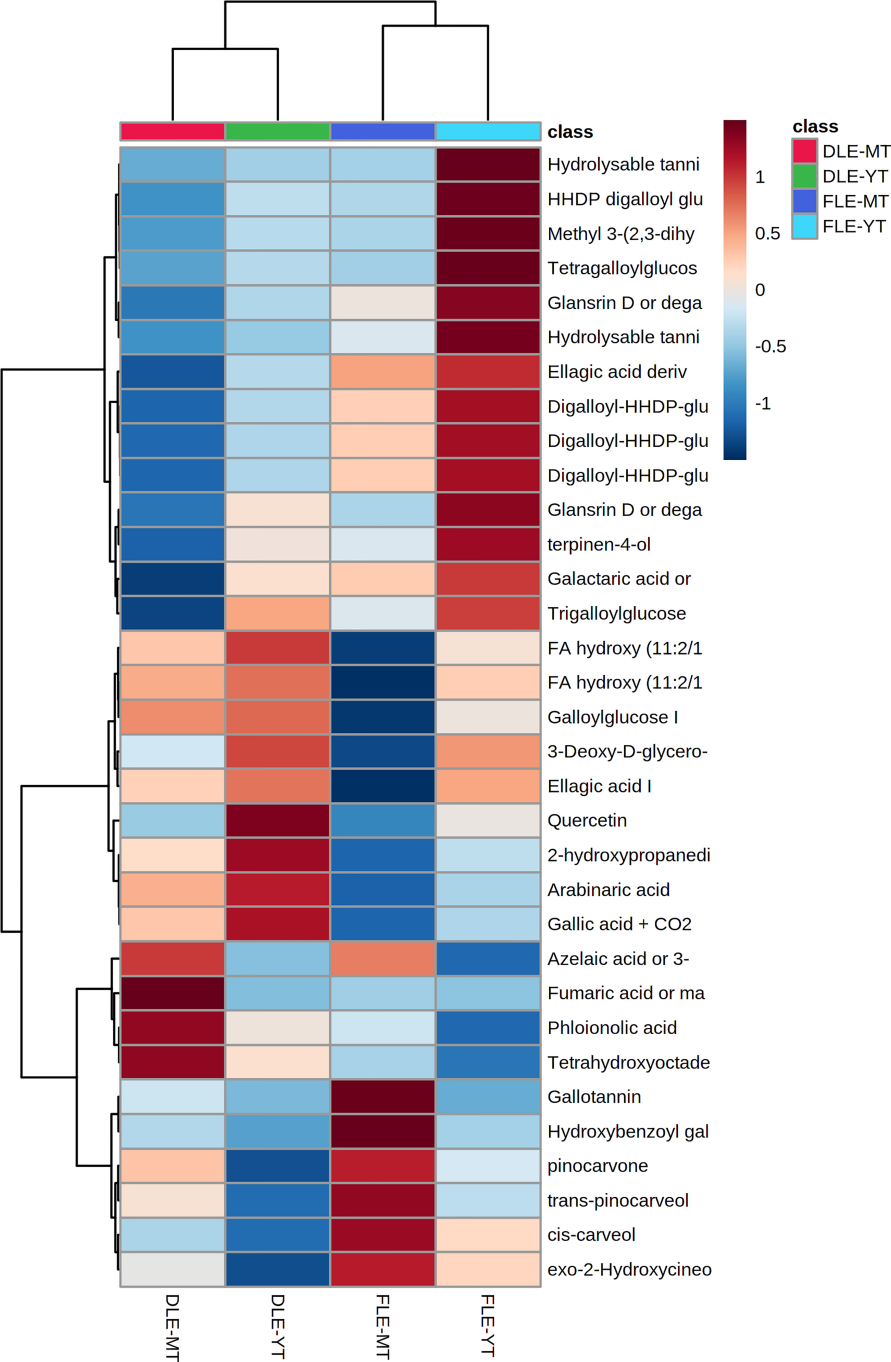
Heatmap showing the distribution of the 33 metabolites affected by both leaf pre-processing and the trees’ maturity between the four groups of samples [aqueous extracts prepared with dried leaves of mature *E. globulus* trees (DLE-MT; red); aqueous extracts prepared with dried leaves of young *E. globulus* trees (DLE-YT; green); aqueous extracts prepared with fresh leaves of mature *E. globulus* trees (FLE-MT; purple), and aqueous extracts prepared with fresh leaves of young *E. globulus* trees (FLE-YT; blue)].

The interaction between the two studied factors induced significant alterations (*p* < 0.05) in 33 identified metabolites (**Table S2**). The distribution of these compounds by the groups of samples is shown in the heatmap of **Figure 6**. Aqueous extracts prepared with fresh leaves of young trees are mainly characterized by the presence of higher contents of ellagitannins (ellagic acid derivatives), while dried leaf extracts of young trees are richer in fatty acids. In turn, fresh leaf extracts of mature trees distinguished from the remaining groups by its greater levels in monoterpenes and terpenoid derivatives, whereas extracts prepared with dried leaves of mature trees showed higher contents of fatty and organic acids. Moreover, dried leaf extracts (of young and mature trees) can be discriminated from fresh ones (aqueous extracts prepared with fresh leaves of young and mature trees) by the higher contents of fatty acids, which had been previously reported in the study of the effect of the leaves’ pre-processing (section 3.2).

## 4 Discussion

This study focused on obtaining the volatile and non-volatile metabolic profile of aqueous extracts prepared with fresh and dried leaves of young and mature *E. globulus* trees. The extracts characterized by GC-MS and HPLC-MS/MS showed a higher diversity of non-volatile compounds when compared with volatile ones. Due to the nature of the employed extraction process, i.e., an extraction in deionized water heated to a temperature below its boiling point, as expected, polar compounds were more easily extracted than non-polar constituents, such as terpenes, which are usually the focus of research studies with *E. globulus*. These non-polar metabolites are, in general, associated with the chemical composition of essential oils of *E. globulus* leaves (Song et al., 2009; Ghaffar et al., 2015). Apart from the present work, the study conducted by Pan et al. (2020) was, to the best of our knowledge, the only analyzing the chemical composition of eucalyptus leaf aqueous extracts by HPLC analysis. However, their extracts were obtained by decoction at room temperature for 24 h, resulting in different diversities of volatile and non-volatile compounds than those presented in this study.

From the universe of metabolites detected in eucalyptus leaf extracts, a clear separation between aqueous extracts prepared with leaves from trees of different maturities and with distinctly processed leaves was observed for both volatile and non-volatile metabolites, indicating that the two studied factors (maturity of the tree and type of leaf pre-processing) influenced the composition of the aqueous extracts (**Figure 2**). These results are corroborated by the outcomes of Silvestre et al. (1997) and Russo et al. (2015), revealing that the chemical composition of juvenile and mature *E. globulus* leaves are different. This supports the hypothesis that changes in the morphology and color of *E. globulus* leaves during the tree development may reflect metabolic variations that occur not only at the level of volatile compounds, but also of non-volatile metabolites, which may represent an adaptive strategy that eucalyptus developed throughout evolution to reduce their susceptibility to herbivore attacks conferred by smaller heights at younger stages (O’Reilly-Wapstra et al., 2007). Although the type of leaf pre-processing was not the main factor behind the differences in the non-volatile composition of the groups of samples, drying the leaf biomass at 60 °C may have significantly affected the pool of volatile metabolites in the aqueous extracts under study (Karabacak et al., 2018). Accordingly, it has been reported that drying plant materials can lead to the loss and change in volatile compounds by several phenomena, such as oxidation and thermal degradation (Karabacak et al., 2018).

Besides the maturity of the tree and the leaf pre-processing, it should be highlighted that the composition of aqueous extracts can also be influenced by other factors such as environmental conditions, soil type, genetics, and the physiological state of the trees (Reigosa et al., 2000; Marinho et al., 2022). As an example, under stressful conditions, such as drought and nutrient deficiency, eucalyptus trees can alter metabolite production, releasing large quantities of them (Reigosa et al., 2000), thus changing the proportions and, possibly, the composition of metabolic features. Therefore, before envisioning any potential application, attention must always be paid to the phytochemical profile of the samples.

### 4.1 Metabolic alterations caused by the leaf pre-processing

Although the drying process can lead to the loss of compounds by thermal and oxidative degradation as stated earlier, it can also enrich the extracts in low molecular weight compounds resulting from those chemical processes. In general, fresh leaf extracts had higher contents of carbohydrates (di- and polysaccharides), condensed tannins, ellagic acid derivatives, hydroxycinnamic acids, and terpenes, when compared with extracts prepared with dried leaves. These metabolites were negatively affected by the drying process that caused the thermal oxidative degradation of some compounds.

The information on the non-structural carbohydrates’ composition of *Eucalyptus* leaves is scarce. The available studies so far determined the content of soluble sugars (fructose, glucose, and sucrose) and starch in dried *E. globulus* leaves (Quentin et al., 2016) and studied the variations of sugar metabolism of different *Eucalyptus* sp. in response to water deficit (Merchant et al., 2006). In the present work, di- and polysaccharides were identified in the extracts as these polymers are easily extracted by hot water (Shi, 2016). The higher levels observed in fresh leaf extracts were probably due to the thermosensitivity of these compounds to the high drying temperature.

Condensed tannins (or proanthocyanidins) as well as ellagic acid derivatives are also affected by temperature variations and the oven-drying process has been reported to highly reduce their extractability from plant leaves (Cork and Krockenberger, 1991; Salminen, 2003). In fact, the content of these phytochemicals in fresh leaf extracts was 83 to 765-fold and 9 to 720-fold higher in fresh leaf extracts than in dried leaf extracts, respectively, as observed by the FC values (**Table 1**). Accordingly, when it comes to hydroxycinnamic acids, although they have been detected in extracts prepared with fresh (Puig et al., 2018; Pan et al., 2020b) and dried leaves (Pombal et al., 2014; Ghareeb et al., 2019) of *E. globulus* trees, in this study, they were also more abundant in extracts prepared with fresh leaves than in dried leaf extracts.

Monoterpenoids are volatile metabolites comprising two linked isoprene units that due to their low molecular weight are characteristic components of plant essential oils, conferring them multiple beneficial properties, like anti-inflammatory, antimicrobial, and antioxidant activities (Zielińska-Błajet and Feder-Kubis, 2020). Although monoterpenoids have been more frequently associated with the composition of extracts and essential oils from fresh eucalyptus leaves [e.g. (Song et al., 2009; Tolba et al., 2015; Yong et al., 2019; Pan et al., 2020a; Sørensen et al., 2020)], their presence has also been reported in dried leaf extracts and essential oils [e.g. (Pombal et al., 2014; Ghaffar et al., 2015; Benchaa et al., 2018)]. In this study, only the content of eucalyptol was found to be significantly higher in fresh leaf extracts than in extracts prepared with dried leaves (**Table 1**).

Terpenoid derivatives like cypellocarpin B, α-limonene diepoxide, and exo-2-hydroxycineole acetate were also significantly more abundant in extracts prepared with fresh leaves than in dried leaf extracts. Cypellocarpin B, being a glucose ester of the monoterpenoid oleuropeic acid, is a secondary metabolite of plants with a widespread occurrence in *Eucalyptus* sp., with important roles in the plant defense and oxidative stress tolerance (Goodger and Woodrow, 2011). Limonene diepoxide and exo-2-hydroxycineole acetate are monoterpenoid derivatives reported as volatiles emitted from fresh leaves of mature *Eucalyptus* sp. (Sørensen et al., 2020). Being derivatives of highly volatile monoterpenes, the higher levels of these compounds in fresh leaf extracts compared with dried leaf extracts could be possibly due to the loss and/or degradation during the oven-drying process.

In opposition to extracts prepared with fresh leaves, dried leaf extracts were characterized by having significantly higher levels of amino acid derivatives, C_13_ norisoprenoids, fatty acid derivatives, and low molecular weight organic acids. It is noticeable that the leaf drying process resulted in the increased content or formation of compounds of lower molecular weight than those obtained in fresh leaf extracts, potentially due to the thermal decomposition of their higher molecular weight precursors (Salminen, 2003). Considering this, amino acid glucosides or deoxypentosides were formed or increased their content due to the oven-drying process that can cause the breakdown of glycopeptides or glycoproteins. Similarly, galactosylglycerol or glucosylglycerol was probably originated by the decomposition of glycerolipids present in the leaves (Guzman et al., 2014). Also, the increased content of tri- and tetra-hydroxylated fatty acids suggests the influence of thermal oxidation of fatty acids from the leaves’ composition caused by their pre-processing. In turn, low molecular weight organic acids found at increased levels in dried leaf extracts may arise as a consequence of the degradation of cell wall carbohydrates induced by the temperature increase in the drying process. It is relevant to highlight the increased contents of arabinaric acid (aldaric acid), and erythronic acid or threonic acid (aldonic acid) that can be formed by the oxidation of aldoses.

The aroma compounds C_13_ norisoprenoids are generated by the decomposition of carotenoids by chemical, photochemical, or enzymatic processes (Kanasawud and Crouzet, 1990). The thermal decomposition of carotenoids (Kanasawud and Crouzet, 1990) explains the results obtained in this work, in which higher levels of 4-hydroxy-4-(3-oxo-1-butenyl)-3,5,5-trimethylcyclohex-2-en-1-one and 3-hydroxy-5,6-epoxy-beta-ionone (**Table 1**) were higher in extracts prepared with dried leaves than in fresh leaf extracts. This last compound has been described by Kurokawa et al. (1998) as having strong allelopathic activities, with high impact on lettuce seed germination. This suggests a high potential interest in dried leaf extracts, especially from young trees, for use as a natural herbicide. In fact, in a recent study, it was revealed that aqueous extracts prepared with dried leaves from young *E. globulus* foliar-applied, twice-a-week at 250 g L^-1^ had potent herbicidal activities against purslane seedlings, used as a model weed species (Pinto et al., 2021). The differences in the content of hydrolysable tannins in the tested extracts were pointed out as a possible cause for the high allelopathic activity of dried *E. globulus* leaf extracts when compared with that of fresh leaf extracts. Considering the results of the present work, the high levels of C_13_ norisoprenoids in aqueous extracts prepared with dried leaves can be also an explanation for the strong herbicidal effect of the dried leaf extracts against those weeds. However, it should be taken into account that the allelopathic activity is not restricted to a single class of compounds, but to a wide range of secondary metabolites (Cheng and Cheng, 2015).

### 4.2 Metabolic alterations induced by the maturity of the tree

Most investigations conducted so far focused on characterizing the chemical composition of *Eucalyptus* leaf extracts or essential oils using biomass from mature trees or fail to mention the maturity of the plant material used. Accordingly, to the best of our knowledge, Heskes et al. (2012) is the only study analyzing the composition of juvenile leaf extracts. However, the authors targeted their research on specific classes, like oleuropeyl glucose esters and flavanones. Still, since eucalyptus leaves exhibit different phytochemical profiles during their developmental stages, studies dealing with the biological potential of *E. globulus* should focus on a comparison between trees at different developmental phases, in order to assess the most effective source of biologically-active compounds.

In this study, the aqueous extracts prepared with leaves from young trees differed from mature ones by having a greater content of benzoic acids, carbohydrates, ellagic and gallic acid derivatives, fatty acid derivatives, flavonoids, and terpenoid derivatives. Additionally, it is relevant to highlight that flavonoids showed to be an important class of compounds in extracts prepared with juvenile leaves, since they contained high contents of glucosides of luteolin, quercetin and kaempferol (**Table 2**). These compounds have powerful reducing properties, mainly responsible for the antioxidant activity of *E. globulus* leaf extracts (Boulekbache-Makhlouf et al., 2013; Puig et al., 2018). In contrast, aqueous extracts prepared with leaves from mature trees were essentially richer in monoterpenoids and organic acids. Therefore, in general, the metabolite diversity decreased with the increase of trees’ maturity (**Table 2**). The differences between juvenile and mature leaf extracts may reflect eucalyptus metabolic adjustments according to the trees’ developmental stage, thus supporting the above-raised hypothesis that due to their larger heights, mature trees do not need to expend as much energy on the synthesis of defense metabolites as young trees to protect themselves from herbivore attacks and ensure the species perpetuation (O’Reilly-Wapstra et al., 2007; Calviño-Cancela and Rubido-Bará, 2013).

Regarding terpenes, the results revealed that oxygenated monoterpenes and terpenoid derivatives were statistically significant for discriminating the trees’ maturity (**Table 2**). Regarding the first class of compounds, extracts from juvenile leaves were richer in terpineol isomers, while the ones prepared with leaves from mature trees had a greater abundance in limonen-1,2-diol, *trans*-pinocarveol, and pinocarvone. Although Silvestre et al. (1997) described that mature eucalyptus leaf essential oils presented higher levels of oxygenated monoterpenes, like terpinen-4-ol and eucalyptol, as well as sesquiterpene alcohols such as α-terpinyl acetate, than those prepared with leaves from young trees, they also demonstrated the high variability of the monoterpene contents of the essential oils with the geographical localization of the trees and the sampling seasons. When it comes to terpenoid derivatives, juvenile leaf extracts detained greater contents of oleuropeic acid (a monoterpenoid acid) glycosides, such as derivatives of oleuropeic and kaempferol (resinoside A or resinoside B), α-terpinyl acetate, and triterpene acid-*O*-hexoside, whereas mature leaf extracts were richer in oleuropeic acid noreugenin derivatives, and cypellocarpin C or eucalmalduside A. All these compounds have been widely found in plants from the Myrtaceae family, including *E. globulus*, and are secondary metabolites with several roles in plants’ defense mechanisms (Goodger and Woodrow, 2011).

### 4.3 Metabolic alterations caused by the leaf pre-processing and the trees’ maturity

As previously stated, both factors are responsible for the metabolomic differences registered in this study. Hence, it was possible to observe that each of the 4 groups of samples has a characteristic metabolomic profile in which some chemical classes can be highlighted (**Figure 6**).

Compared with the other 2 groups of samples, aqueous extracts prepared with fresh leaves of young and mature trees showed considerably higher levels of hydrolysable tannins, i.e., derivatives of gallic and ellagic acids (**Figure 6**) than dried leaf extracts of young and mature trees. However, their diversity and quantity were found to be greater in young trees than in mature ones, indicating that the trees’ maturity is the factor mainly affecting the presence of these metabolites in eucalyptus leaves, as was registered above in the study of the effect of the trees’ maturity on the extracts’ chemical composition (**Table 2**). Hydrolysable tannins represent about 40% of the total phytochemicals present in the bark and leaves of forest trees and are known to have a strong allelopathic activity, affecting not only nutrient decomposition rate, soil microorganisms, and enzyme activities, but also plant’s photosynthetic system (Flamini, 2012). Furthermore, aqueous extracts prepared with dried leaves of mature trees presented a particularly relevant profile of increased contents of monoterpenes and terpenoid derivatives.

The metabolomic fraction of aqueous extracts prepared with dried leaves of young and mature trees revealed to be very distinct from each other and from fresh leaf extracts (**Figure 6**). In general, it was possible to conclude that dried leaf extracts were characterized by having greater levels of fatty and low molecular weight organic acids associated with the degradation of high molecular weight compounds, such as long-chain fatty acids and polysaccharides, as previously discussed. In fact, this conclusion is also supported by the results presented in **Table 1** which shows that these two phytochemical classes are statistically significant for leaf pre-processing discrimination.

The present study paved the way for the characterization of the volatile and non-volatile composition of aqueous extracts prepared with leaves from young *E. globulus* trees and for its comparison with the metabolic features of mature eucalyptus leaf extracts. The results revealed that both factors, the maturity of the trees and the leaf pre-processing, influenced the chemical composition of the analyzed extracts. Considering both factors, fresh leaf extracts of young eucalyptus trees had a greater content of ellagic and gallic acid derivatives, whereas extracts prepared with fresh leaves of mature trees showed higher contents of monoterpenes and terpenoid derivatives. In turn, aqueous extracts prepared with dried leaves of young trees had a higher content of fatty acids, while dried leaf extracts of mature *E. globulus* were richer in organic acids.

This knowledge will encourage the use of the leaf biomass of young *E. globulus* trees, besides mature ones, thus contributing to a better spatial planning, since this species often presents an invasive behavior in several European countries. Furthermore, the results of this study show that the type of compounds recovered from eucalyptus leaf extracts and, consequently, the resulting beneficial effects depend on the chosen leaf pre-processing and maturity of the trees from which the leaves are harvested. Accordingly, while extracts prepared with fresh leaves of young trees could be used as anti-inflammatories, anti-carcinogens, or as alternatives to synthetic fertilizers due to their content of phenolic compounds and terpenes, those prepared with dried leaves could represent strong candidates for eco-friendly herbicides and antimicrobials due the high levels of C_13_ norisoprenoids. In turn, the phytochemical composition of mature trees’ fresh leaf extracts rich in terpenes could justify their use as bioherbicides, and antimicrobial, antioxidant, anti-carcinogenic, neuroprotective, and hypoglycemic agents.

## Data availability statement

The original contributions presented in the study are included in the article/**Supplementary Material**. Further inquiries can be directed to the corresponding author.

## Author contributions

MP designed and carried out the experiments and contributed to data analysis and writing. CS contributed to experimental design and writing. RP funding, conceptualization, and manuscript revision. JR provided funding and contributed to writing. FF provided funding, conceived the project, contributed to experimental design, and revised the manuscript. IV conducted data analysis and contribute to writing. All authors contributed to the article and approved the submitted version.

## Funding

We gratefully acknowledge the financial support from Fundação para a Ciência e Tecnologia (FCT) through the research project ref. PCIF/GVB/0150/2018 and through “Verão com Ciência 2020 - Hands on Science for Sustainable AgriFood Production: From the Soil to the Fork” project. MP acknowledges FCT for providing a PhD scholarship (2021.07342.BD). CS acknowledges FCT for providing a PhD scholarship (SFRH/BD/115643/2016). Thanks are also due to GreenUPorto through UIDB/05748/2020 and UIDP/05748/2020 (FCT/MCTES), and REQUIMTE though UIDB/50006/2020. IV acknowledges the funding program (DL 57/2016 – Norma transitória) supported by FCT.

## Conflict of interest

The authors declare that the research was conducted in the absence of any commercial or financial relationships that could be construed as a potential conflict of interest.

## Publisher’s note

All claims expressed in this article are solely those of the authors and do not necessarily represent those of their affiliated organizations, or those of the publisher, the editors and the reviewers. Any product that may be evaluated in this article, or claim that may be made by its manufacturer, is not guaranteed or endorsed by the publisher.
