# Supplementary material for: Untargeted metabolomic profiling of fresh and dried leaf extracts of young and mature Eucalyptus globulus trees indicates differences in the presence of specialized metabolites
Source: Front Plant Sci. 2022 Nov 14;13:986197. doi: 10.3389/fpls.2022.986197 (PMC9702574; doi:10.3389/fpls.2022.986197)
Supplement: Supplementary file 1 [file DataSheet_1.docx]

Supplementary Material

# Supplementary Tables

**Table S1.** HPLC and MS detector conditions used for the analysis of aqueous extracts of *E. globulus* leaves

| **HPLC conditions** | |
| --- | --- |
| **Column** | Gemini C_18_ column (150 mm × 4.6 mm; 3 μm particle size) and guard column (4 mm × 3.0 mm) from Phenomenex (Torrance, CA) |
| **Column temperature** | 29ºC |
| **Mobile phase** | (A) methanol; (B) 0.1% aqueous formic acid |
| **Gradient** | 0-40 min 10%-30% A; 40-60 min 30%-45% A; 60-90 min 45%-100% A; 90-95 min 100% A; 95-110 min 100%-10% A; 110-120 min 10% A |
| **Flow rate** | 0.45 mL min^-1^ |
| **Injection volume** | 20 µL |
| **MS conditions (negative mode)** | |
| **Spray voltage** | 2500 V |
| **Sheat gas (N_2_) flow** | 50 arbitrary units |
| **Auxiliary gas (N_2_) flow** | 10 arbitrary units |
| **Ion transfer tube temperature** | 300ºC |
| **Vaporizer temperature** | 350ºC |
| **Orbitrap resolution (Master Scan)** | 60000 |
| **Orbitrap resolution (MS^n^ Scan)** | 15000 |
| **Mass range** | m/z 100 - 1000 |

**Table S2.** Identification of the metabolites with respective chemical classes significant (*p* < 0.05) for discrimination of the samples by the trees' maturity and the leaf pre-processing. The results were obtained by two-way ANOVA comparing the metabolomic profile of the 4 groups of samples by GC-MS and HPLC-MS analyses (*p* < 0.05 are marked in colour; compounds simultaneously affected by both factors are marked in bold).

|  |  |  | ***Adjusted p-values*** | |
| --- | --- | --- | --- | --- |
| **Analysis** | **Metabolite** | **Class** | **Trees’ maturity** | **Leaf processing** |
| LC-MS | Glutamine | Amino acid or derivative | 0.13 | 0.02 |
| LC-MS | Methylserine hexoside | Amino acid or derivative | 0.22 | <0.01 |
| LC-MS | N-1-Deoxy-1-fructosyl)alanine | Amino acid or derivative | 0.51 | <0.01 |
| LC-MS | Serine glucoside | Amino acid or derivative | 0.76 | <0.01 |
| LC-MS | **Methyl 3-(2,3-dihydroxy-3-methylbutyl)-4-hydroxybenzoate / isopropyl 2-hydroxy-3-(3-hydroxy-4-methoxyphenyl)propanoate / isopropyl 2-hydroxy-3-(4-hydroxy-3-methoxyphenyl)propanoate VII** | **Benzoic acid** | <0.01 | <0.01 |
| LC-MS | Dihydroxybenzoic acid pentoside derivative | Benzoic acid | 0.20 | <0.01 |
| LC-MS | Methyl 3-(2,3-dihydroxy-3-methylbutyl)-4-hydroxybenzoate or isopropyl 2-hydroxy-3-(3-hydroxy-4-methoxyphenyl)propanoate or isopropyl 2-hydroxy-3-(4-hydroxy-3-methoxyphenyl)propanoate I | Benzoic acid | 0.05 | 0.29 |
| LC-MS | Methyl 3-(2,3-dihydroxy-3-methylbutyl)-4-hydroxybenzoate or isopropyl 2-hydroxy-3-(3-hydroxy-4-methoxyphenyl)propanoate or isopropyl 2-hydroxy-3-(4-hydroxy-3-methoxyphenyl)propanoate II | Benzoic acid | 0.02 | 0.72 |
| LC-MS | Methyl 3-(2,3-dihydroxy-3-methylbutyl)-4-hydroxybenzoate or isopropyl 2-hydroxy-3-(3-hydroxy-4-methoxyphenyl)propanoate or isopropyl 2-hydroxy-3-(4-hydroxy-3-methoxyphenyl)propanoate III | Benzoic acid | 0.02 | 0.73 |
| LC-MS | Protocatechuic acid | Benzoic acid | 0.05 | <0.01 |
| GC-MS | 3-Hydroxy-5, 6-epoxy-beta-ionone | C13 norisoprenoids | 0.43 | 0.01 |
| GC-MS | 4-Hydroxy-4-(3-oxo-1-butenyl)-3,5,5-trimethylcyclohex-2-en-1-one | C13 norisoprenoids | 0.22 | 0.01 |
| LC-MS | 3-(2,3-Dihydroxypropoxy)-2-hydroxypropyl galactopyranoside | Carbohydrate or derivative | 0.02 | 0.11 |
| LC-MS | **3-Deoxy-D-glycero-D-galacto-2-nonulosonic acid** | **Carbohydrate or derivative** | <0.01 | 0.01 |
| LC-MS | Galactitol or Sorbitol or Mannitol or Iditol | Carbohydrate or derivative | 0.01 | 0.14 |
| LC-MS | Hexose polymer | Carbohydrate or derivative | 0.64 | <0.01 |
| LC-MS | Melezitose or raffinose or maltotriose | Carbohydrate or derivative | 0.27 | 0.01 |
| LC-MS | Monosaccharide II | Carbohydrate or derivative | 0.17 | <0.01 |
| LC-MS | Monosaccharide I | Carbohydrate or derivative | 0.21 | 0.03 |
| LC-MS | Monosaccharide IV | Carbohydrate or derivative | 0.07 | 0.04 |
| LC-MS | Oligosaccharide | Carbohydrate or derivative | 0.73 | <0.01 |
| LC-MS | Sulphated carbohydrate | Carbohydrate or derivative | 0.03 | 0.88 |
| LC-MS | Turanose or palatinose or maltose or lactose or sucrose or trehalose | Carbohydrate or derivative | 0.89 | <0.01 |
| LC-MS | (+)-Epigallocatechin | Condensed tannin | 0.89 | <0.01 |
| LC-MS | (epi)catechin-(epi)catechingallate I | Condensed tannin | 0.71 | 0.01 |
| LC-MS | (epi)catechin-(epi)gallocatechin | Condensed tannin | 0.32 | <0.01 |
| LC-MS | Procyanidin B-type I | Condensed tannin | 0.46 | <0.01 |
| LC-MS | Procyanidin B-type II | Condensed tannin | 0.43 | <0.01 |
| LC-MS | Procyanidin B-type IV | Condensed tannin | 0.67 | <0.01 |
| LC-MS | Procyanidin C-type | Condensed tannin | 0.68 | <0.01 |
| LC-MS | 2',3'-Bis-O-degalloyl rugosin F isomer I | Ellagic acid or derivative | 0.04 | 0.41 |
| LC-MS | 2',3'-Bis-O-degalloyl rugosin F isomer II | Ellagic acid or derivative | 0.33 | 0.02 |
| LC-MS | **Digalloyl-HHDP-gluconic acid I** | **Ellagic acid or derivative** | 0.04 | <0.01 |
| LC-MS | **Digalloyl-HHDP-gluconic acid II** | **Ellagic acid or derivative** | 0.04 | <0.01 |
| LC-MS | **Digalloyl-HHDP-gluconic acid III** | **Ellagic acid or derivative** | 0.04 | <0.01 |
| LC-MS | Dehydro-galloyl-HHDP-hexoside | Ellagic acid or derivative | 0.01 | 0.21 |
| LC-MS | **Ellagic acid derivative II** | **Ellagic acid or derivative** | 0.02 | <0.01 |
| LC-MS | **Ellagic acid I** | **Ellagic acid or derivative** | 0.01 | 0.03 |
| LC-MS | **Glansrin D or degalloyl rugosin F isomer I** | **Ellagic acid or derivative** | <0.01 | 0.02 |
| LC-MS | Digalloyl-HHDP-gluconic acid IV | Ellagic acid or derivative | 0.05 | <0.01 |
| LC-MS | Digalloyl-HHDP-gluconic acid V | Ellagic acid or derivative | 0.05 | <0.01 |
| LC-MS | Digalloyl-HHDP-gluconic acid VII | Ellagic acid or derivative | 0.13 | <0.01 |
| LC-MS | di-HHDP-glucose IV | Ellagic acid or derivative | 0.03 | 0.99 |
| LC-MS | di-HHDP-glucose IX | Ellagic acid or derivative | <0.01 | 0.42 |
| LC-MS | di-HHDP-glucose V | Ellagic acid or derivative | 0.04 | 0.34 |
| LC-MS | Ellagic acid derivative I | Ellagic acid or derivative | 0.40 | 0.01 |
| LC-MS | **Glansrin D or degalloyl rugosin F isomer II** | **Ellagic acid or derivative** | <0.01 | <0.01 |
| LC-MS | Ellagic acid derivative III | Ellagic acid or derivative | 0.39 | 0.02 |
| LC-MS | **HHDP digalloyl glucose II** | **Ellagic acid or derivative** | <0.01 | <0.01 |
| LC-MS | Eucalbanin C I | Ellagic acid or derivative | 0.07 | <0.01 |
| LC-MS | **Hydrolysable tannin I** | **Ellagic acid or derivative** | <0.01 | <0.01 |
| LC-MS | **Hydrolysable tannin III** | **Ellagic acid or derivative** | <0.01 | <0.01 |
| LC-MS | HHDP galloyl glucose I | Ellagic acid or derivative | 0.03 | 0.71 |
| LC-MS | HHDP galloyl glucose III | Ellagic acid or derivative | 0.03 | 0.76 |
| LC-MS | HHDP galloyl glucose IV | Ellagic acid or derivative | 0.02 | 0.06 |
| LC-MS | HHDP galloyl glucose VII | Ellagic acid or derivative | 0.03 | 0.14 |
| LC-MS | Hydrolysable tannin II | Ellagic acid or derivative | 0.02 | 0.14 |
| LC-MS | tri-*O*-methylellagic acid | Ellagic acid or derivative | 0.01 | 0.42 |
| LC-MS | (3S,4R)-3,4,5-trihydroxy-2-oxo-pentanoic acid | Fatty acid or derivative | 0.03 | 0.84 |
| LC-MS | 6,8-dihydroxy-octanoic acid | Fatty acid or derivative | 0.20 | <0.01 |
| LC-MS | 9,12,13-trihydroxy-10,15-octadecadienoic acid | Fatty acid or derivative | 0.04 | 0.25 |
| LC-MS | DGGA(28:4;O) | Fatty acid or derivative | <0.01 | 0.90 |
| LC-MS | **FA hydroxy (11:2/11:2) I** | **Fatty acid or derivative** | 0.03 | 0.01 |
| LC-MS | FA hydroxy (11:2/11:2) III | Fatty acid or derivative | 0.30 | <0.01 |
| LC-MS | **FA hydroxy (11:2/11:2) II** | **Fatty acid or derivative** | 0.04 | 0.02 |
| LC-MS | **Phloionolic acid** | **Fatty acid or derivative** | <0.01 | <0.01 |
| LC-MS | Galactosylglycerol or Glucosylglycerol | Fatty acid or derivative | 0.06 | <0.01 |
| LC-MS | **Tetrahydroxyoctadecenoic acid** | **Fatty acid or derivative** | <0.01 | <0.01 |
| LC-MS | Avicularin or guajavarin I | Flavonoid | 0.01 | 0.16 |
| LC-MS | Avicularin or guajavarin II | Flavonoid | <0.01 | 0.37 |
| LC-MS | Catechin | Flavonoid | 0.96 | 0.01 |
| LC-MS | **Quercetin** | **Flavonoid** | <0.01 | <0.01 |
| LC-MS | Kaempferol 3-glucuronide | Flavonoid | 0.02 | 0.47 |
| LC-MS | Kaempferol-3-O-rutinoside | Flavonoid | 0.01 | 0.72 |
| LC-MS | Luteolin 7-O-glucuronide | Flavonoid | <0.01 | 0.54 |
| LC-MS | Quercetin 3-O-glucuronide | Flavonoid | 0.02 | 0.15 |
| LC-MS | Quercetin-4'-O-glucoside or quercetin-3-glucoside (isoquercitrin) | Flavonoid | <0.01 | 0.92 |
| LC-MS | Digalloylglucose I | Gallic acid or derivative | 0.10 | 0.01 |
| LC-MS | Digalloylglucose II | Gallic acid or derivative | 0.05 | 0.61 |
| LC-MS | Digalloylglucose III | Gallic acid or derivative | 0.03 | 0.98 |
| LC-MS | Digalloylglucose IV | Gallic acid or derivative | 0.72 | 0.01 |
| LC-MS | Digalloylglucose V | Gallic acid or derivative | 0.82 | 0.01 |
| LC-MS | Digalloylglucose VI | Gallic acid or derivative | 0.01 | 0.34 |
| LC-MS | Gallic acid derivative | Gallic acid or derivative | 0.05 | 0.57 |
| LC-MS | Gallic acid pentoside | Gallic acid or derivative | 0.10 | <0.01 |
| LC-MS | Galloyl glucose derivative I | Gallic acid or derivative | 0.02 | 0.08 |
| LC-MS | Galloyl glucose derivative III | Gallic acid or derivative | 0.05 | 0.54 |
| LC-MS | Galloyl glucose derivative X | Gallic acid or derivative | 0.23 | 0.01 |
| LC-MS | **Gallic acid + CO_2_** | **Gallic acid or derivative** | 0.04 | <0.01 |
| LC-MS | Galloylglucose II | Gallic acid or derivative | 0.54 | <0.01 |
| LC-MS | Galloylshikimic acid I | Gallic acid or derivative | 0.01 | 0.69 |
| LC-MS | Galloylshikimic acid II | Gallic acid or derivative | 0.01 | 0.68 |
| LC-MS | Galloylshikimic acid III | Gallic acid or derivative | 0.01 | 0.66 |
| LC-MS | **Gallotannin** | **Gallic acid or derivative** | <0.01 | <0.01 |
| LC-MS | **Galloylglucose I** | **Gallic acid or derivative** | 0.01 | <0.01 |
| LC-MS | **Hydroxybenzoyl galloyl glucoside** | **Gallic acid or derivative** | <0.01 | <0.01 |
| LC-MS | **Tetragalloylglucose I** | **Gallic acid or derivative** | 0.02 | 0.03 |
| LC-MS | **Trigalloylglucose II** | **Gallic acid or derivative** | <0.01 | 0.03 |
| LC-MS | hydroxyjasmonic acid sulfate III | Gallic acid or derivative | <0.01 | 0.60 |
| LC-MS | Tetragalloylglucose II | Gallic acid or derivative | 0.02 | 0.24 |
| LC-MS | Tetragalloylglucose III | Gallic acid or derivative | 0.01 | 0.13 |
| LC-MS | Tetragalloylglucose IV | Gallic acid or derivative | 0.01 | 0.29 |
| LC-MS | Trigalloylglucose I | Gallic acid or derivative | 0.01 | 0.95 |
| LC-MS | Trigalloylglucose III | Gallic acid or derivative | 0.01 | 0.10 |
| LC-MS | Trigalloylglucose V | Gallic acid or derivative | <0.01 | 0.10 |
| LC-MS | Trigalloylglucose VI | Gallic acid or derivative | 0.04 | 0.60 |
| LC-MS | Trigalloylglucose VII | Gallic acid or derivative | 0.03 | 0.42 |
| LC-MS | *cis*-5-caffeoylquinic acid | Hydroxycinnamic acid | 0.05 | 0.01 |
| LC-MS | *cis*-5-O-p-coumaroylquinic acid | Hydroxycinnamic acid | 0.04 | 0.21 |
| LC-MS | Coumaroyl hexose | Hydroxycinnamic acid | 0.63 | 0.03 |
| LC-MS | Sinapoyl glucoside I | Hydroxycinnamic acid | 0.54 | <0.01 |
| LC-MS | *trans*-5-caffeoylquinic acid | Hydroxycinnamic acid | 0.34 | 0.01 |
| LC-MS | Mallophenol B or Macarangioside B III | Megastigmane derivative | 0.04 | 0.68 |
| GC-MS | alpha-terpineol_21.94 min | Monoterpenoid | 0.06 | 0.05 |
| GC-MS | alpha-terpineol_23.17 min | Monoterpenoid | 0.04 | 0.10 |
| GC-MS | eucalyptol | Monoterpenoid | 0.16 | 0.03 |
| GC-MS | ***cis*-carveol** | **Monoterpenoid** | 0.01 | <0.01 |
| GC-MS | Limonene-1,2-diol | Monoterpenoid | 0.01 | 0.70 |
| GC-MS | Linalool | Monoterpenoid | 0.01 | 0.37 |
| GC-MS | **pinocarvone** | **Monoterpenoid** | 0.01 | 0.03 |
| GC-MS | **terpinen-4-ol** | **Monoterpenoid** | <0.01 | <0.01 |
| GC-MS | ***trans*-pinocarveol** | **Monoterpenoid** | <0.01 | 0.01 |
| LC-MS | 2-methylcitric acid or homocitric acid I | Organic acid | 0.41 | 0.01 |
| LC-MS | 3-dehydroshikimic acid | Organic acid | 0.01 | 0.42 |
| LC-MS | Aconitic acid I | Organic acid | 0.01 | 0.08 |
| LC-MS | Aconitic acid II | Organic acid | 0.01 | 0.60 |
| LC-MS | Arabinonic acid or D-Lyxonic acid or D-ribulose or Ribonic acid or Xylonic acid II | Organic acid | 0.05 | 0.05 |
| LC-MS | Erythronic acid or Threonic acid I | **Organic acid** | 0.29 | <0.01 |
| LC-MS | Erythronic acid or Threonic acid II | Organic acid | 0.86 | 0.01 |
| LC-MS | isocitric acid | Organic acid | 0.16 | <0.01 |
| LC-MS | **2-hydroxypropanedioic acid** | **Organic acid** | 0.04 | 0.01 |
| LC-MS | Shikimic acid | Organic acid | <0.01 | 0.54 |
| LC-MS | Tartaric acid or meso-tartaric acid I | Organic acid | 0.04 | 0.27 |
| LC-MS | Tartaric acid or meso-tartaric acid II | Organic acid | 0.36 | <0.01 |
| LC-MS | **Arabinaric acid** | **Organic acid** | 0.02 | <0.01 |
| LC-MS | **Azelaic acid or 3-methylsuberic acid** | **Organic acid** | <0.01 | <0.01 |
| LC-MS | **Fumaric acid or maleic acid** | **Organic acid** | <0.01 | <0.01 |
| LC-MS | **Galactaric acid or Glucaric acid II** | **Organic acid** | 0.03 | 0.02 |
| LC-MS | Citrinin | Other compounds | 0.05 | 0.42 |
| GC-MS | dihydroactinidiolide | Other compounds | 0.17 | <0.01 |
| GC-MS | (E)-*p*-2,8-menthadien-1-ol | Terpenoid derivative | 0.64 | <0.01 |
| GC-MS | alpha-Limonene diepoxide | Terpenoid derivative | 0.05 | 0.01 |
| GC-MS | alpha-terpinyl acetate | Terpenoid derivative | <0.01 | 0.76 |
| GC-MS | beta-eudesmol | Terpenoid derivative | 0.04 | 0.61 |
| GC-MS | *cis*-linalool oxide | Terpenoid derivative | <0.01 | 0.19 |
| LC-MS | Cypellocarpin A or eucaglobulin or Globulusin B or Eucalmaidin B VIII | Terpenoid derivative | 0.02 | 0.44 |
| LC-MS | Cypellocarpin B | Terpenoid derivative | 0.66 | 0.02 |
| LC-MS | Cypellocarpin C or Eucalmalduside A I | Terpenoid derivative | 0.02 | 0.42 |
| GC-MS | *endo*-borneol | Terpenoid derivative | 0.43 | 0.04 |
| LC-MS | Resinoside A or Resinoside B | Terpenoid derivative | 0.04 | 0.21 |
| GC-MS | *trans*-linalool oxide | Terpenoid derivative | 0.01 | 0.16 |
| GC-MS | **exo-2-Hydroxycineole acetate** | **Terpenoid derivative** | <0.01 | <0.01 |
| LC-MS | Triterpene acid-O-hexoside II | Terpenoid derivative | 0.02 | 0.24 |

# Supplementary Figures

| **A** |
| --- |
| 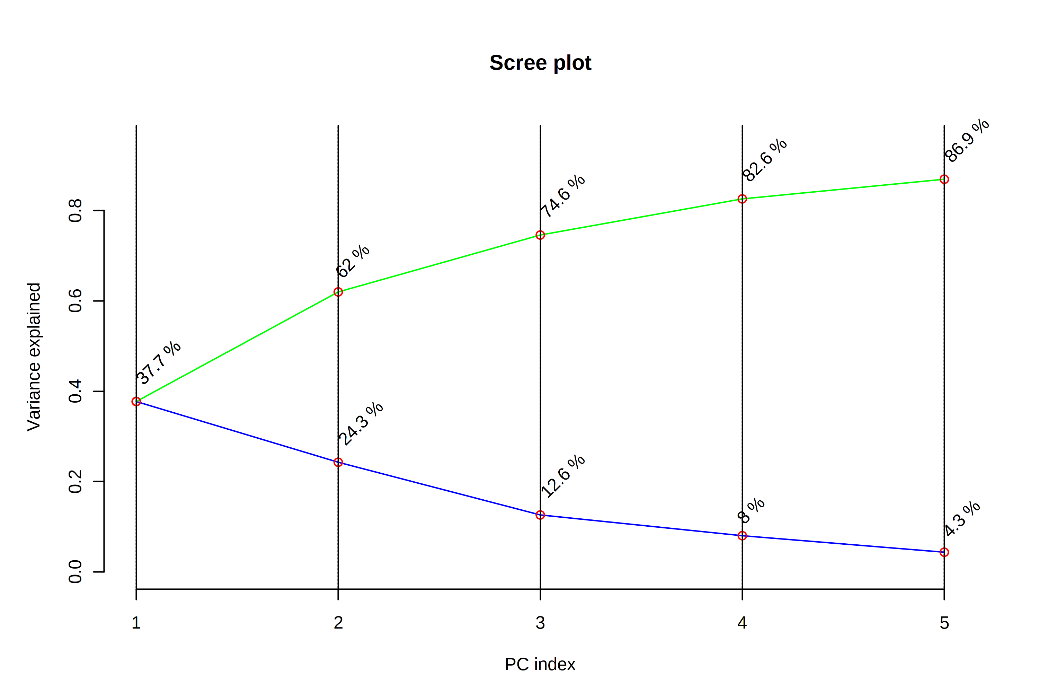 |
| **B** |
| 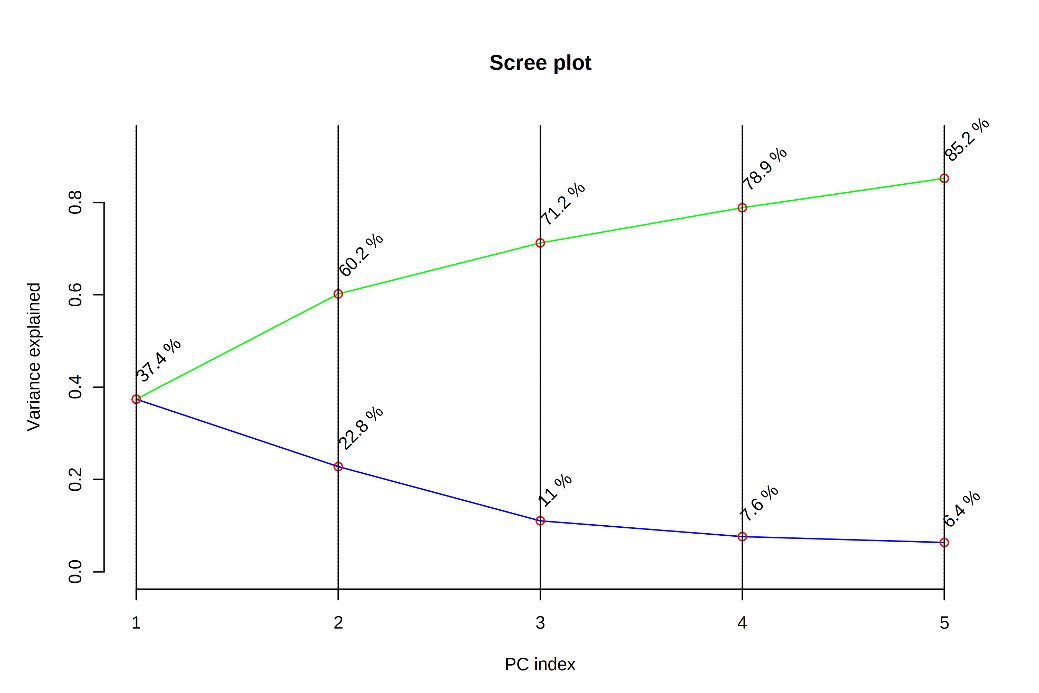 |

**Figure S1.** Scree plots of the Principal Components Analysis (PCA) for the results of (A) GC-MS and (B) LC-MS analyses of the *Eucalyptus globulus* leaf extracts.
